# Supplementary material for: Pyruvate Dehydrogenase Kinase 1 inhibition mediated oxidative phosphorylation enhancement in cartilage promotes osteoarthritis progression
Source: BMC Musculoskelet Disord. 2023 Jul 20;24:597. doi: 10.1186/s12891-023-06585-6 (PMC10357736; doi:10.1186/s12891-023-06585-6)
Supplement: Supplementary file 1 — Additional file 1. [file 12891_2023_6585_MOESM1_ESM.zip › BP.Gsea.1653623667859/GOBP_NEGATIVE_REGULATION_OF_MRNA_METABOLIC_PROCESS.html]

Details for gene set GOBP\_NEGATIVE\_REGULATION\_OF\_MRNA\_METABOLIC\_PROCESS[GSEA]

|  || Dataset | 55457\_collapsed\_to\_symbols.cls.cls #OA\_versus\_normal.cls.cls #OA\_versus\_normal\_repos |
| Phenotype | cls.cls#OA\_versus\_normal\_repos |
| Upregulated in class | normal |
| GeneSet | GOBP\_NEGATIVE\_REGULATION\_OF\_MRNA\_METABOLIC\_PROCESS |
| Enrichment Score (ES) | -0.47727033 |
| Normalized Enrichment Score (NES) | -1.9659233 |
| Nominal p-value | 0.0 |
| FDR q-value | 0.038597908 |
| FWER p-Value | 0.411 |
Table: GSEA Results Summary

  

Fig 1: Enrichment plot: GOBP\_NEGATIVE\_REGULATION\_OF\_MRNA\_METABOLIC\_PROCESS      
 Profile of the Running ES Score & Positions of GeneSet Members on the Rank Ordered List

  

| SYMBOL | TITLE | RANK IN GENE LIST | RANK METRIC SCORE | RUNNING ES | CORE ENRICHMENT || 1 | SRSF9 | serine and arginine rich splicing factor 9 [Source:HGNC Symbol;Acc:HGNC:10791] | 882 | 0.548 | -0.0485 | No |
| 2 | SAP18 | Sin3A associated protein 18 [Source:HGNC Symbol;Acc:HGNC:10530] | 1099 | 0.496 | -0.0470 | No |
| 3 | NBAS | NBAS subunit of NRZ tethering complex [Source:HGNC Symbol;Acc:HGNC:15625] | 1132 | 0.490 | -0.0314 | No |
| 4 | NELFE | negative elongation factor complex member E [Source:HGNC Symbol;Acc:HGNC:13974] | 1135 | 0.489 | -0.0134 | No |
| 5 | TRAF3IP2 | TRAF3 interacting protein 2 [Source:HGNC Symbol;Acc:HGNC:1343] | 1939 | 0.368 | -0.0624 | No |
| 6 | MAPK14 | mitogen-activated protein kinase 14 [Source:HGNC Symbol;Acc:HGNC:6876] | 2352 | 0.320 | -0.0827 | No |
| 7 | ANGEL2 | angel homolog 2 [Source:HGNC Symbol;Acc:HGNC:30534] | 2608 | 0.290 | -0.0919 | No |
| 8 | APOBEC1 | apolipoprotein B mRNA editing enzyme catalytic subunit 1 [Source:HGNC Symbol;Acc:HGNC:604] | 2695 | 0.280 | -0.0882 | No |
| 9 | BARD1 | BRCA1 associated RING domain 1 [Source:HGNC Symbol;Acc:HGNC:952] | 3051 | 0.245 | -0.1069 | No |
| 10 | C1QBP | complement C1q binding protein [Source:HGNC Symbol;Acc:HGNC:1243] | 3083 | 0.241 | -0.1004 | No |
| 11 | TENT5A | terminal nucleotidyltransferase 5A [Source:HGNC Symbol;Acc:HGNC:18345] | 3204 | 0.230 | -0.1012 | No |
| 12 | CIRBP | cold inducible RNA binding protein [Source:HGNC Symbol;Acc:HGNC:1982] | 3441 | 0.207 | -0.1119 | No |
| 13 | TENT5C | terminal nucleotidyltransferase 5C [Source:HGNC Symbol;Acc:HGNC:24712] | 3646 | 0.190 | -0.1208 | No |
| 14 | A1CF | APOBEC1 complementation factor [Source:HGNC Symbol;Acc:HGNC:24086] | 4368 | 0.123 | -0.1725 | No |
| 15 | PCBP4 | poly(rC) binding protein 4 [Source:HGNC Symbol;Acc:HGNC:8652] | 4577 | 0.107 | -0.1847 | No |
| 16 | PTBP1 | polypyrimidine tract binding protein 1 [Source:HGNC Symbol;Acc:HGNC:9583] | 4650 | 0.100 | -0.1867 | No |
| 17 | HNRNPD | heterogeneous nuclear ribonucleoprotein D [Source:HGNC Symbol;Acc:HGNC:5036] | 4830 | 0.085 | -0.1975 | No |
| 18 | PAIP1 | poly(A) binding protein interacting protein 1 [Source:HGNC Symbol;Acc:HGNC:16945] | 4851 | 0.083 | -0.1960 | No |
| 19 | ZC3H14 | zinc finger CCCH-type containing 14 [Source:HGNC Symbol;Acc:HGNC:20509] | 4896 | 0.080 | -0.1964 | No |
| 20 | DAZ1 | deleted in azoospermia 1 [Source:HGNC Symbol;Acc:HGNC:2682] | 4920 | 0.078 | -0.1953 | No |
| 21 | E2F1 | E2F transcription factor 1 [Source:HGNC Symbol;Acc:HGNC:3113] | 5137 | 0.061 | -0.2099 | No |
| 22 | PKP1 | plakophilin 1 [Source:HGNC Symbol;Acc:HGNC:9023] | 5248 | 0.052 | -0.2166 | No |
| 23 | EIF4ENIF1 | eukaryotic translation initiation factor 4E nuclear import factor 1 [Source:HGNC Symbol;Acc:HGNC:16687] | 5330 | 0.045 | -0.2212 | No |
| 24 | HNRNPA0 | heterogeneous nuclear ribonucleoprotein A0 [Source:HGNC Symbol;Acc:HGNC:5030] | 5485 | 0.033 | -0.2320 | No |
| 25 | PKP3 | plakophilin 3 [Source:HGNC Symbol;Acc:HGNC:9025] | 5510 | 0.032 | -0.2327 | No |
| 26 | RBM42 | RNA binding motif protein 42 [Source:HGNC Symbol;Acc:HGNC:28117] | 6059 | -0.009 | -0.2751 | No |
| 27 | DAZL | deleted in azoospermia like [Source:HGNC Symbol;Acc:HGNC:2685] | 6221 | -0.022 | -0.2868 | No |
| 28 | CTR9 | "CTR9 homolog, Paf1/RNA polymerase II complex component [Source:HGNC Symbol;Acc:HGNC:16850]" | 6430 | -0.037 | -0.3017 | No |
| 29 | MYD88 | MYD88 innate immune signal transduction adaptor [Source:HGNC Symbol;Acc:HGNC:7562] | 6638 | -0.052 | -0.3159 | No |
| 30 | RBM10 | RNA binding motif protein 10 [Source:HGNC Symbol;Acc:HGNC:9896] | 6647 | -0.053 | -0.3145 | No |
| 31 | RNF40 | ring finger protein 40 [Source:HGNC Symbol;Acc:HGNC:16867] | 6681 | -0.054 | -0.3151 | No |
| 32 | SLC11A1 | solute carrier family 11 member 1 [Source:HGNC Symbol;Acc:HGNC:10907] | 7153 | -0.092 | -0.3484 | No |
| 33 | ELAVL1 | ELAV like RNA binding protein 1 [Source:HGNC Symbol;Acc:HGNC:3312] | 7426 | -0.113 | -0.3655 | No |
| 34 | HNRNPC | heterogeneous nuclear ribonucleoprotein C [Source:HGNC Symbol;Acc:HGNC:5035] | 7437 | -0.114 | -0.3620 | No |
| 35 | DYRK1A | dual specificity tyrosine phosphorylation regulated kinase 1A [Source:HGNC Symbol;Acc:HGNC:3091] | 7667 | -0.132 | -0.3750 | No |
| 36 | TARDBP | TAR DNA binding protein [Source:HGNC Symbol;Acc:HGNC:11571] | 7864 | -0.146 | -0.3849 | No |
| 37 | TOB1 | "transducer of ERBB2, 1 [Source:HGNC Symbol;Acc:HGNC:11979]" | 7892 | -0.147 | -0.3816 | No |
| 38 | FMR1 | FMRP translational regulator 1 [Source:HGNC Symbol;Acc:HGNC:3775] | 7944 | -0.151 | -0.3800 | No |
| 39 | RNPS1 | RNA binding protein with serine rich domain 1 [Source:HGNC Symbol;Acc:HGNC:10080] | 8275 | -0.177 | -0.3992 | No |
| 40 | TRAF2 | TNF receptor associated factor 2 [Source:HGNC Symbol;Acc:HGNC:12032] | 8322 | -0.180 | -0.3961 | No |
| 41 | HNRNPA2B1 | heterogeneous nuclear ribonucleoprotein A2/B1 [Source:HGNC Symbol;Acc:HGNC:5033] | 8576 | -0.200 | -0.4084 | No |
| 42 | RBMX | RNA binding motif protein X-linked [Source:HGNC Symbol;Acc:HGNC:9910] | 8938 | -0.227 | -0.4282 | No |
| 43 | VIP | vasoactive intestinal peptide [Source:HGNC Symbol;Acc:HGNC:12693] | 9070 | -0.237 | -0.4296 | No |
| 44 | YBX1 | Y-box binding protein 1 [Source:HGNC Symbol;Acc:HGNC:8014] | 9623 | -0.285 | -0.4621 | No |
| 45 | IKBKE | inhibitor of nuclear factor kappa B kinase subunit epsilon [Source:HGNC Symbol;Acc:HGNC:14552] | 9646 | -0.287 | -0.4532 | No |
| 46 | HNRNPU | heterogeneous nuclear ribonucleoprotein U [Source:HGNC Symbol;Acc:HGNC:5048] | 9847 | -0.306 | -0.4575 | No |
| 47 | TENT4A | terminal nucleotidyltransferase 4A [Source:HGNC Symbol;Acc:HGNC:16705] | 10003 | -0.322 | -0.4577 | No |
| 48 | TRAF5 | TNF receptor associated factor 5 [Source:HGNC Symbol;Acc:HGNC:12035] | 10085 | -0.329 | -0.4518 | No |
| 49 | ELAVL4 | ELAV like RNA binding protein 4 [Source:HGNC Symbol;Acc:HGNC:3315] | 10412 | -0.362 | -0.4639 | Yes |
| 50 | GDNF | glial cell derived neurotrophic factor [Source:HGNC Symbol;Acc:HGNC:4232] | 10475 | -0.370 | -0.4550 | Yes |
| 51 | CSDE1 | cold shock domain containing E1 [Source:HGNC Symbol;Acc:HGNC:29905] | 10559 | -0.379 | -0.4475 | Yes |
| 52 | DHX9 | DExH-box helicase 9 [Source:HGNC Symbol;Acc:HGNC:2750] | 10627 | -0.386 | -0.4384 | Yes |
| 53 | HNRNPAB | heterogeneous nuclear ribonucleoprotein A/B [Source:HGNC Symbol;Acc:HGNC:5034] | 10632 | -0.386 | -0.4244 | Yes |
| 54 | SRSF6 | serine and arginine rich splicing factor 6 [Source:HGNC Symbol;Acc:HGNC:10788] | 10659 | -0.389 | -0.4121 | Yes |
| 55 | SRSF10 | serine and arginine rich splicing factor 10 [Source:HGNC Symbol;Acc:HGNC:16713] | 10926 | -0.422 | -0.4172 | Yes |
| 56 | SRSF7 | serine and arginine rich splicing factor 7 [Source:HGNC Symbol;Acc:HGNC:10789] | 10930 | -0.422 | -0.4019 | Yes |
| 57 | LARP1 | "La ribonucleoprotein 1, translational regulator [Source:HGNC Symbol;Acc:HGNC:29531]" | 10964 | -0.425 | -0.3887 | Yes |
| 58 | CDK9 | cyclin dependent kinase 9 [Source:HGNC Symbol;Acc:HGNC:1780] | 11071 | -0.441 | -0.3806 | Yes |
| 59 | SYNCRIP | synaptotagmin binding cytoplasmic RNA interacting protein [Source:HGNC Symbol;Acc:HGNC:16918] | 11182 | -0.454 | -0.3724 | Yes |
| 60 | METTL16 | "methyltransferase 16, N6-methyladenosine [Source:HGNC Symbol;Acc:HGNC:28484]" | 11346 | -0.478 | -0.3674 | Yes |
| 61 | NOCT | nocturnin [Source:HGNC Symbol;Acc:HGNC:14254] | 11566 | -0.510 | -0.3657 | Yes |
| 62 | LARP4B | La ribonucleoprotein 4B [Source:HGNC Symbol;Acc:HGNC:28987] | 11695 | -0.531 | -0.3560 | Yes |
| 63 | SUPT5H | "SPT5 homolog, DSIF elongation factor subunit [Source:HGNC Symbol;Acc:HGNC:11469]" | 11735 | -0.539 | -0.3391 | Yes |
| 64 | YBX3 | Y-box binding protein 3 [Source:HGNC Symbol;Acc:HGNC:2428] | 12024 | -0.599 | -0.3394 | Yes |
| 65 | DHX34 | DExH-box helicase 34 [Source:HGNC Symbol;Acc:HGNC:16719] | 12040 | -0.603 | -0.3182 | Yes |
| 66 | MAPKAPK2 | MAPK activated protein kinase 2 [Source:HGNC Symbol;Acc:HGNC:6887] | 12094 | -0.619 | -0.2995 | Yes |
| 67 | U2AF2 | U2 small nuclear RNA auxiliary factor 2 [Source:HGNC Symbol;Acc:HGNC:23156] | 12226 | -0.652 | -0.2856 | Yes |
| 68 | DND1 | DND microRNA-mediated repression inhibitor 1 [Source:HGNC Symbol;Acc:HGNC:23799] | 12266 | -0.667 | -0.2639 | Yes |
| 69 | HNRNPK | heterogeneous nuclear ribonucleoprotein K [Source:HGNC Symbol;Acc:HGNC:5044] | 12341 | -0.688 | -0.2442 | Yes |
| 70 | SECISBP2 | SECIS binding protein 2 [Source:HGNC Symbol;Acc:HGNC:30972] | 12416 | -0.712 | -0.2237 | Yes |
| 71 | THRAP3 | thyroid hormone receptor associated protein 3 [Source:HGNC Symbol;Acc:HGNC:22964] | 12426 | -0.717 | -0.1978 | Yes |
| 72 | CCNT1 | cyclin T1 [Source:HGNC Symbol;Acc:HGNC:1599] | 12517 | -0.757 | -0.1769 | Yes |
| 73 | PABPC1 | poly(A) binding protein cytoplasmic 1 [Source:HGNC Symbol;Acc:HGNC:8554] | 12562 | -0.778 | -0.1515 | Yes |
| 74 | FUS | FUS RNA binding protein [Source:HGNC Symbol;Acc:HGNC:4010] | 12739 | -0.902 | -0.1319 | Yes |
| 75 | SFSWAP | splicing factor SWAP [Source:HGNC Symbol;Acc:HGNC:10790] | 12747 | -0.908 | -0.0988 | Yes |
| 76 | SRSF4 | serine and arginine rich splicing factor 4 [Source:HGNC Symbol;Acc:HGNC:10786] | 12749 | -0.911 | -0.0652 | Yes |
| 77 | TAF15 | TATA-box binding protein associated factor 15 [Source:HGNC Symbol;Acc:HGNC:11547] | 12828 | -1.023 | -0.0334 | Yes |
| 78 | ZFP36 | ZFP36 ring finger protein [Source:HGNC Symbol;Acc:HGNC:12862] | 12846 | -1.052 | 0.0042 | Yes |
Table: GSEA details [plain text format]

  

Fig 2: GOBP\_NEGATIVE\_REGULATION\_OF\_MRNA\_METABOLIC\_PROCESS      
 Blue-Pink O' Gram in the Space of the Analyzed GeneSet

  

Fig 3: GOBP\_NEGATIVE\_REGULATION\_OF\_MRNA\_METABOLIC\_PROCESS: Random ES distribution      
 Gene set null distribution of ES for **GOBP\_NEGATIVE\_REGULATION\_OF\_MRNA\_METABOLIC\_PROCESS**
